# HOXA13 promotes high glucose-induced trophoblast cell growth and migration during gestational diabetes by regulating the smad2 pathway

**DOI:** 10.1186/s41065-025-00542-0

**Published:** 2025-08-28

**Authors:** Fei Zhao, Xinhong Liu, Hong Qin

**Affiliations:** Department of Obstetrics and Gynecology, Shenzhen Longhua District Maternal and Child Health Hospital, No. 68, Huawang Road, Longhua District, Shenzhen, 570105 China

**Keywords:** Gestational diabetes mellitus, HOXA13, Migration, Smad2, Trophoblast cell

## Abstract

**Background:**

Gestational diabetes mellitus (GDM) is considered the most common complication of pregnancy, and is a very dangerous disease for both mother and baby. Homeobox A13 (HOXA13) has been discovered to join into some diseases through exhibiting regulatory functions. Importantly, hypermethylation of HOXA13 has been observed in the placental tissues of preeclampsia. However, the regulatory impacts of HOXA13 on GDM progression keep dimness.

**Methods:**

The GDM cell model and GDM rat model were established. The expressions of genes were measured through RT-qPCR, western blot or IHC assay. The cell proliferation was tested through MTT and Edu assays. The cell migration was determined through Transwell assay. The fasting blood glucose of rats was detected through the blood glucose meter.

**Results:**

HOXA13 was verified to be lowly expressed in placental tissues from GDM patients. In addition, the cell proliferation and migration abilities of trophoblast cells were attenuated after high glucose (HG) treatment, but these impacts were counteracted after HOXA13 overexpression. It was further demonstrated that HOXA13 affected the proliferation and migration abilities of HG-triggered trophoblast cells by enhancing smad2 expression. At last, it was testified that HOXA13 can ameliorate GDM symptoms in vivo.

**Conclusion:**

This study manifested that HOXA13 accelerated HG-triggered trophoblast cell growth and migration by regulating the smad2 pathway. This discovery hinted that HOXA13 may be a target for ameliorating GDM.

**Supplementary Information:**

The online version contains supplementary material available at 10.1186/s41065-025-00542-0.

## Introduction

Gestational diabetes mellitus (GDM) is a highly prevalent complication of pregnancy, which is featured by abnormal glucose tolerance and highly fasting blood glucose (≥ 92 mg/dL), leading to persistent high blood sugar levels and jeopardizing the well-being of individuals [1]. During pregnancy, the placental trophoblast cells, known for their vitality, play a crucial role in facilitating proper placental exchange. Any malfunction in these cells can lead to disrupted placental exchange and increased inflammation,, which may generate badly pregnancy outcomes [2]. The development of trophoblast cells is pivotal for successful pregnancy, and take part into the pathophysiology of various pregnancy diseases [3]. As a result, it has become essential to identify valuable targets for regulating trophoblast cells in the progression of gestational diabetes mellitus (GDM).

Homeobox (HOX) genes consist of a group of transcription factors that play a crucial role in various physiological processes, particularly in embryonic development [4]. Interestingly, our previous study has demonstrated that a member of the HOX family-HOXC8 exhibits lower expression in GDM placental tissues and accelerates the proliferation, migration and angiogenesis capacities of high glucose (HG)-evoked trophoblast cells [5]. HOXA13 has been affirmed to belong to the HOX family, and affect the progression of diversiform diseases. As an illustration, HOXA13 influences the FAK/Src pathway to worsen the advancement of gastric cancer [6]. Moreover, HOXA13 impacts the WNT pathway to facilitate tumorigenesis in colon cancer progression [7]. HOXA13 also modulatesBMP-7 expression to accelerate liver regeneration [8]. Furthermore, in human pregnant myometrium, HOXA13 can modulate phenotype regionalization [9]. Additionally, HOXA13 plays a role in enhancing functions related to albumin-induced epithelial-mesenchymal transition by regulating the glucocorticoid receptor signaling pathway [10]. Importantly, hypermethylation of HOXA13 has been existed in the placental tissues of preeclampsia [11]. Nevertheless, the regulatory effects of HOXA13 on the progression of GDM remain unclear.

In this work, it was disclosed that HOXA13 intensified HG-triggered trophoblast cell growth and migration by regulating the smad2 pathway in GDM. This discovery may afford novel views for the treatment of GDM.

## Materials and methods

### Tissue samples

The placental tissues were obtained from normal pregnancy (NP) women (*n* = 16) and GDM patients (*n* = 20). All participators have signed the informed consent. The selection of GDM patients was on the basis of the International Association for Pregnancy Diabetes Research (IADPSG). This work was endorsed by the ethics committee of Shenzhen Longhua District Maternal and Child Health Hospital (Approval No.2023-28).

### Cell lines and treatment

The trophoblast cells (HTR8/SVneo) were obtained from American Type Culture Collection (ATCC, Manassas, VA, USA). The RPMI-1640 medium (Gibco, Carlsbad, CA, USA) with 10% FBS (Gibco, USA) was utilized for cell incubation, and under one humidified incubator (5% CO_2_, 37 °C).

The high glucose (HG, 25 mM, 24 h) was utilized for treating trophoblast cells to mimic GDM cell model [12–15].

### GDM rat model

The female Wistar rats (200–250 g; total *n* = 24, *n* = 6/group) were obtained from Vital River Laboratory Animal Technology Co., Ltd. (Beijing, China). Rats were provided with food and water ad libitum under a 12-hour light-dark cycle. This work was approved by the Animal Care and Use Committee of Shenzhen Longhua District Maternal and Child Health Hospital (Approval No.2023 − 171). On the 6th day of pregnancy, the streptozotocin (STZ, 60 mg/kg, Sigma, USA) were administered to induce the rat model of GDM in the pregnant female rats [5, 16, 17]. After 48 h, the rats of diabetes mellitus were confirmed through blood glucose level exceeding 200 mg/dL. The AAV-Vector or AAV-HOXA13 plasmids were shotted into GDM rats. Placental tissues were obtained for further experiments by sacrificing the rats after a period of 20 days. The rats (*n* = 6/group) were randomly split into the Control, GDM, GDM + AAV-Vector and GDM + AAV-HOXA13 groups.

### Transfection

The pcDNA3.1 vectors targeting HOXA13 (HOXA13) as well as its negative control (Vector), AAV-HOXA13 and its corresponding negative control AAV-Vector were produced from GenePharma (Shanghai, China). The transfection was administered under Lipofectamine 3000 reagent (Invitrogen).

### RT-qPCR

The RNAs isolated from placenta tissues were executed through the TRIzol reagents (Invitrogen, Carlsbad, CA). The cDNAs were generated through the PrimeScript™ RT Master Mix kit (Takara, Dalian, China). The SYBR Premix Ex Taq™ kit (Takara, Shanghai, China) was utilized for administering qPCR. The mRNA expression of HOXA13 was inspected through the 2^−ΔΔCt^ method.

The primer sequences:

HOXA13 forward, 5′-CTCCCCACCTCTGGAAGTC-3′, and reverse, 5′-TTCGTAGCGTATTCCCGTTC-3′;

GAPDH forward, 5′-CAATGACCCCTTCATTGACC-3′, and reverse, 5′-GACAAGCTTCCCGTTCTCA-3′;

### Western blot

Under the RIPA lysis buffer (Thermo Fisher Scientific, Inc.), proteins were gained from placenta tissues or trophoblast cells. Through 10% SDS-PAGE, these proteins were segregated, and then moving them to PVDF membranes (Beyotime, Shanghai, China). After membrane blocking with 5% nonfat-milk, the primary antibodies were appended for 12 h: HOXA13 (1:1000; ab172570, Abcam, Shanghai, China), smad2 (1:2000, ab40855) and β-actin (1 µg/mL, ab8226). Then, the secondary antibodies (1:2000, ab7090) were incubated for an additional 2 h. Finally, the blots were examined using the chemiluminescence detection kit (Thermo Fisher Scientific, Inc.).

### IHC assay

The placenta tissues (paraffin-embedded) were cut into 4 μm slices, and performed with deparaffinization and rehydration. After being sealed with 5% nonfat-milk, the sections were mixed with HOXA13 or Ki67 antibody (overnight, 4 °C), next secondary antibody (1:500, ab6112, Abcam, Shanghai, China) was further appended. The staining was conducted using diaminobenzidine (DAB) followed by restaining with hematoxylin. Under one microscope (Nikon, Tokyo, Japan), IHC images were gained.

### MTT assay

In the 96-well plate, HTR8/SVneo cells (1000 cells/well) were placed. After 24 h, each well was mixed with MTT solution (20 µL, Sigma-Aldrich, St. Louis, MO, USA). Following a 4-hour incubation, DMSO (150 µL, Sigma-Aldrich) was added to dissolve the formazan crystals. Finally, cell viability was confirmed at 570 nm under the microplate reader (Bio-Rad, Hercules, CA, United States),.

### Edu assay

The EdU kit (RiboBio, Guangzhou, China) was utilized. The EdU (50 µM) solution was appended for 2 h incubation. Post permeabilization and fixation, the staining under 4’, 6-diamidi-diamidino-2-phenylindole (DAPI) and Apollo dye solution was made. Finally, under the fluorescence microscopy (Leica, Wetzlar, Germany), the Edu-positive cells (%) were figured up.

### Transwell assay

The upper Transwell chambers (8 µM; Corning, NY, United States) was appended with the serum-free medium containing HTR8/SVneo cells (200 µL). The lower compartments were supplemented with medium containing 20% FBS (600 µL). Post 24 h, moved cells were treated with fixation (4% paraformaldehyde) and dye (0.1% crystal violet). Eventually, the migrated cells were counted under one microscope (Leica, Wetzlar, Germany).

### CO-IP assay

The interaction between HOXA3 and smad2 was verified through the CO-IP kit (Abison Biotechnology Co. Ltd, China). Proteins were mixed with MG-132 (10 µM, proteasome inhibitor). Proteins were co-incubated with primary antibodies. Next, protein A/G plus-agarose beads (Santa Cruz Biotechnology, Santa Cruz, CA, USA) were further supplemented. Post washing, the isolated immunoprecipitation complexes were examined through western blot.

### Detection of fasting blood glucose

The FreeStyle Lite blood glucose meter (Abbot Diabetes Care, Inc.) was used to measure the fasting blood glucose level.

### Statistical analysis

The data were expressed as mean ± standard deviation (SD). The statistical analysis was proceeded with GraphPad Prism 9.0 (GraphPad, La Jolla, USA). The comparisons were estimated under the Student’s t-test, multiple comparisons or one-way ANOVA. The *p* < 0.05 was accepted statistically significant.

## Results

### HOXA13 was lowly expressed in placental tissues from GDM patients

**Fig. 1:**
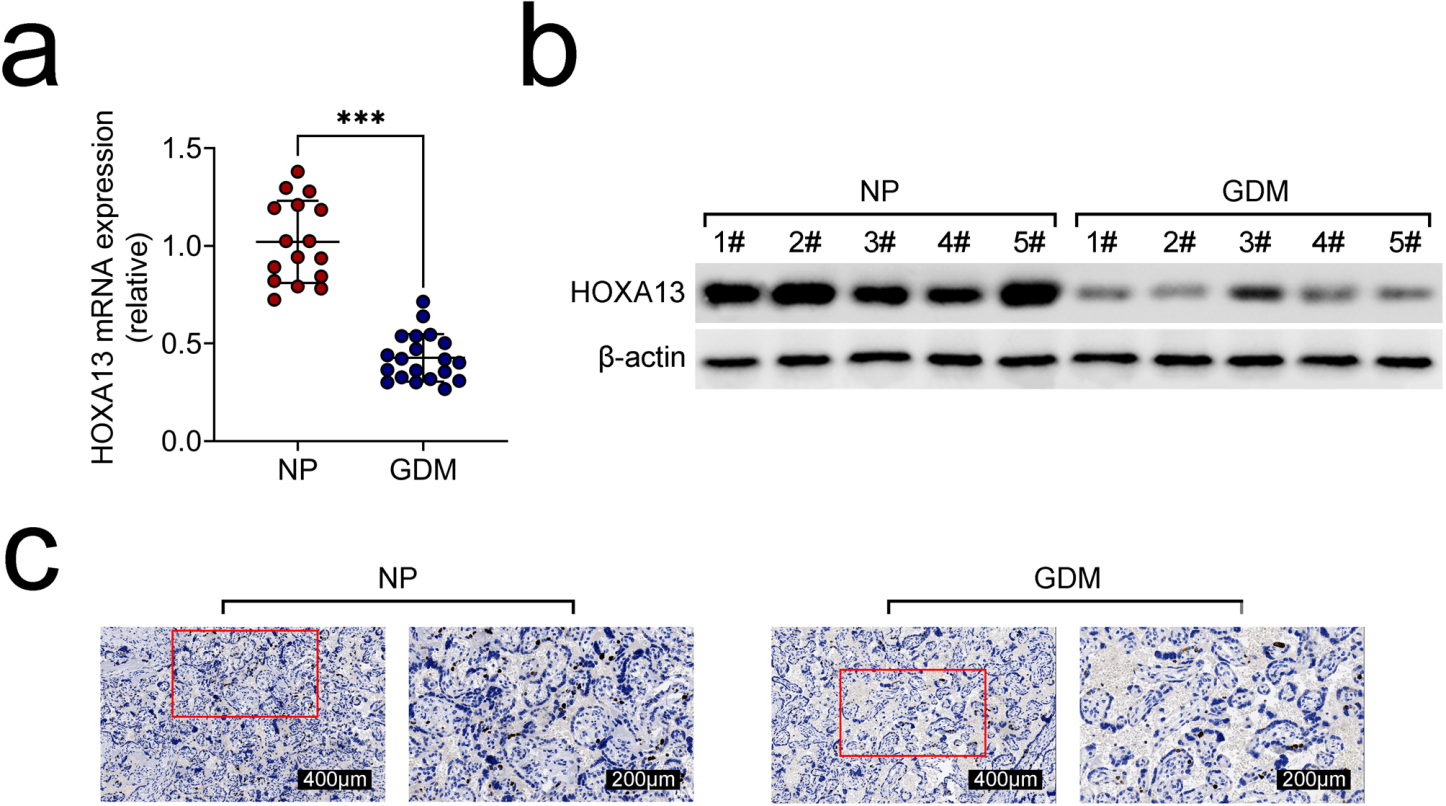
HOXA13 was lowly expressed in placental tissues from GDM patients. (**a**) The mRNA expression of HOXA13 in NP (n = 16) and GDM (n = 20) placental tissues through RT-qPCR. (**b**) The protein expression of HOXA13 in NP (n = 5) and GDM (n = 5) placental tissues through western blot. (**c**) The protein expression of HOXA13 in NP and GDM placental tissues through IHC assay. ***p < 0.001

The mRNA expression of HOXA13 in GDM placental tissues was declined (Fig. 1a). Furthermore, the HOXA13 protein expression was found to be reduced in the GDM group when compared with the NP group. (Fig. 1b). Through IHC assay, it was affirmed that the Ki67 expression was descended in GDM placental tissues (Fig. 1c). In general, HOXA13 owned the declined expression in placental tissues from GDM patients.

### HOXA13 facilitated the proliferation and migration abilities of HG-triggered trophoblast cells

**Fig. 2:**
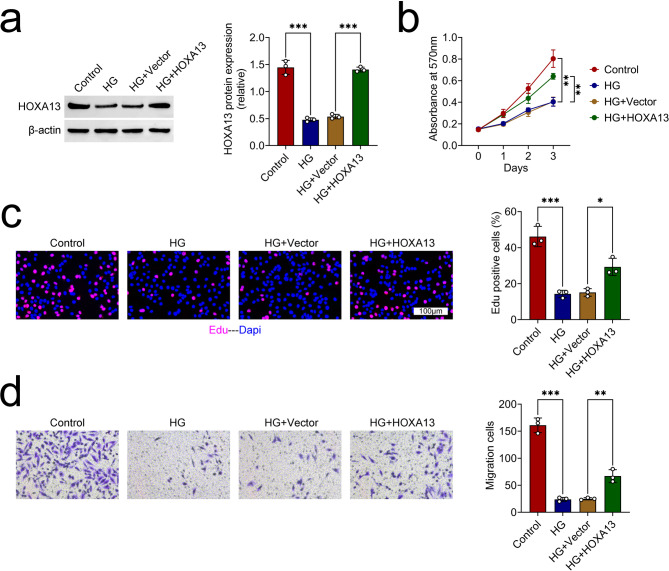
HOXA13 facilitated the proliferation and migration abilities of HG-triggered trophoblast cells. Groups were separated into the Control, HG, HG + Vector and HG + HOXA13 group. (**a**) The protein expression of HOXA13 was examined through western blot. (**b**) The cell viability was tested through MTT assay. (**c**) The cell proliferation was measured through Edu assay. (**d**) The cell migration was determined through Transwell assay. *p < 0.05, **p < 0.01, ***p < 0.001

The HOXA13 protein expression was decreased in HG-triggered trophoblast cells, but this trend was inverted following the HOXA13 overexpression (Fig. 2a). The cell viability was attenuated after HG treatment, but this change was offset after HOXA13 amplification (Fig. 2b). Additionally, cell proliferation was weakened after HG treatment, but this impact was counteracted after HOXA13 overexpression (Fig. 2c). Following treatment with high glucose, the capacity for cell migration decreased, however, this effect was counteracted by the enhancement of HOXA13. (Fig. 2d). Taken together, HOXA13 facilitated the proliferation and migration abilities of HG-triggered trophoblast cells.

### HOXA13 affected the proliferation and migration abilities of HG-triggered trophoblast cells by enhancing smad2 expression

**Fig. 3:**
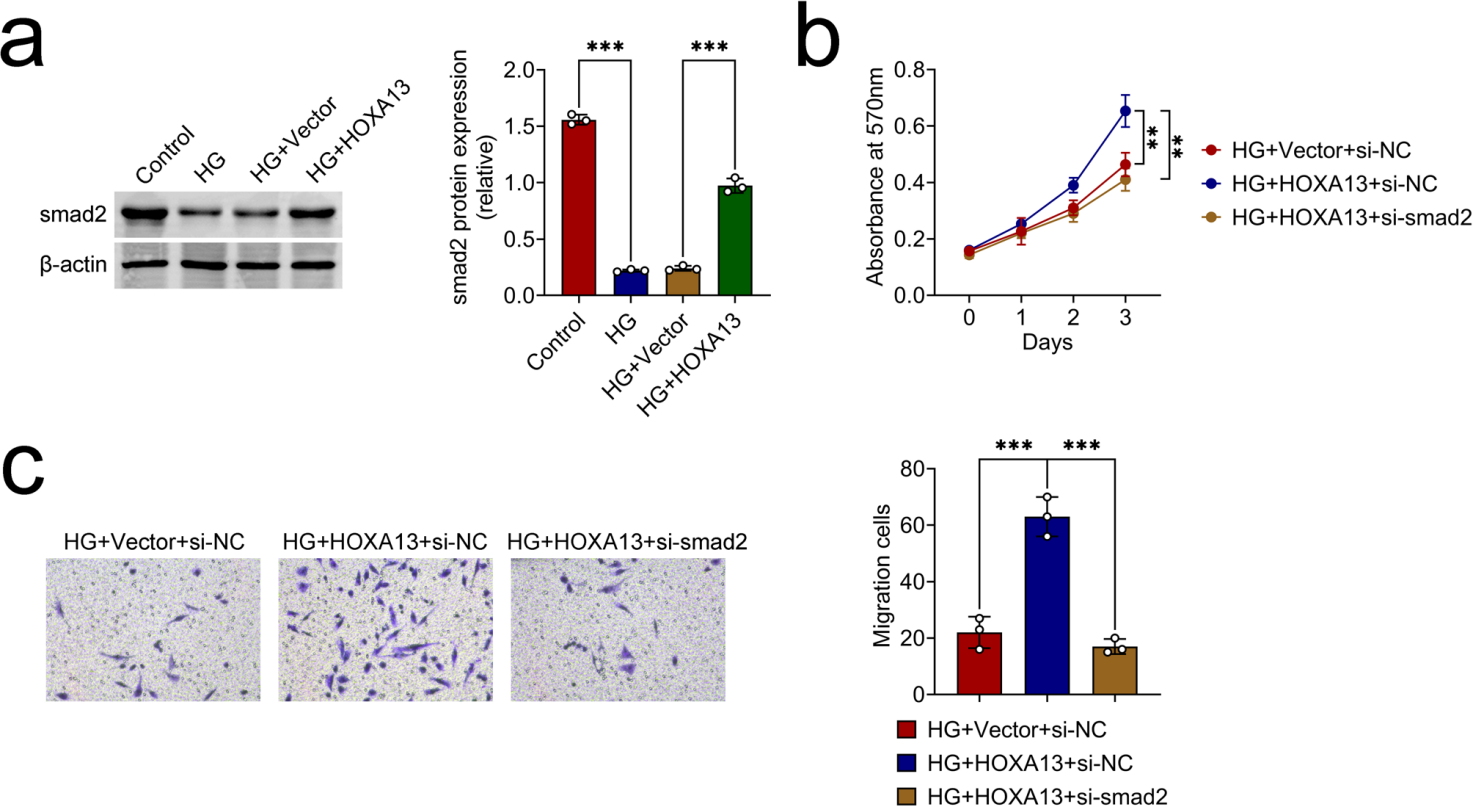
HOXA13 affected the proliferation and migration abilities of HG-triggered trophoblast cells by enhancing smad2 expression (**a**) The protein expression of smad2 was assessed through western blot. Groups were separated into the Control, HG, HG + Vector and HG + HOXA13 group. (**b**) The cell viability was confirmed through MTT assay. Groups were separated into the HG, HG + HOXA13 + si-NC and HG + HOXA13 + si-smad2 group. (**c**) The cell migration was verified through Transwell assay. Groups were separated into the HG, HG + HOXA13 + si-NC and HG + HOXA13 + si-smad2 group. **p < 0.01, ***p < 0.001

The smad2 protein expression was declined after HG treatment, but this impact was neutralized after HOXA13 overexpression (Fig. 3a). Furthermore, the heightened cell viability mediated by HOXA13 overexpression was lessened after smad2 knockdown (Fig. 3b). The enhanced cell migration capability facilitated by the increase in HOXA13 expression was diminished following the inhibition of smad2. (Fig. 3c). To sum up, HOXA13 affected the proliferation and migration abilities of HG-triggered trophoblast cells by enhancing smad2 expression.

### HOXA13 can ameliorate GDM symptoms in vivo

**Fig. 4:**
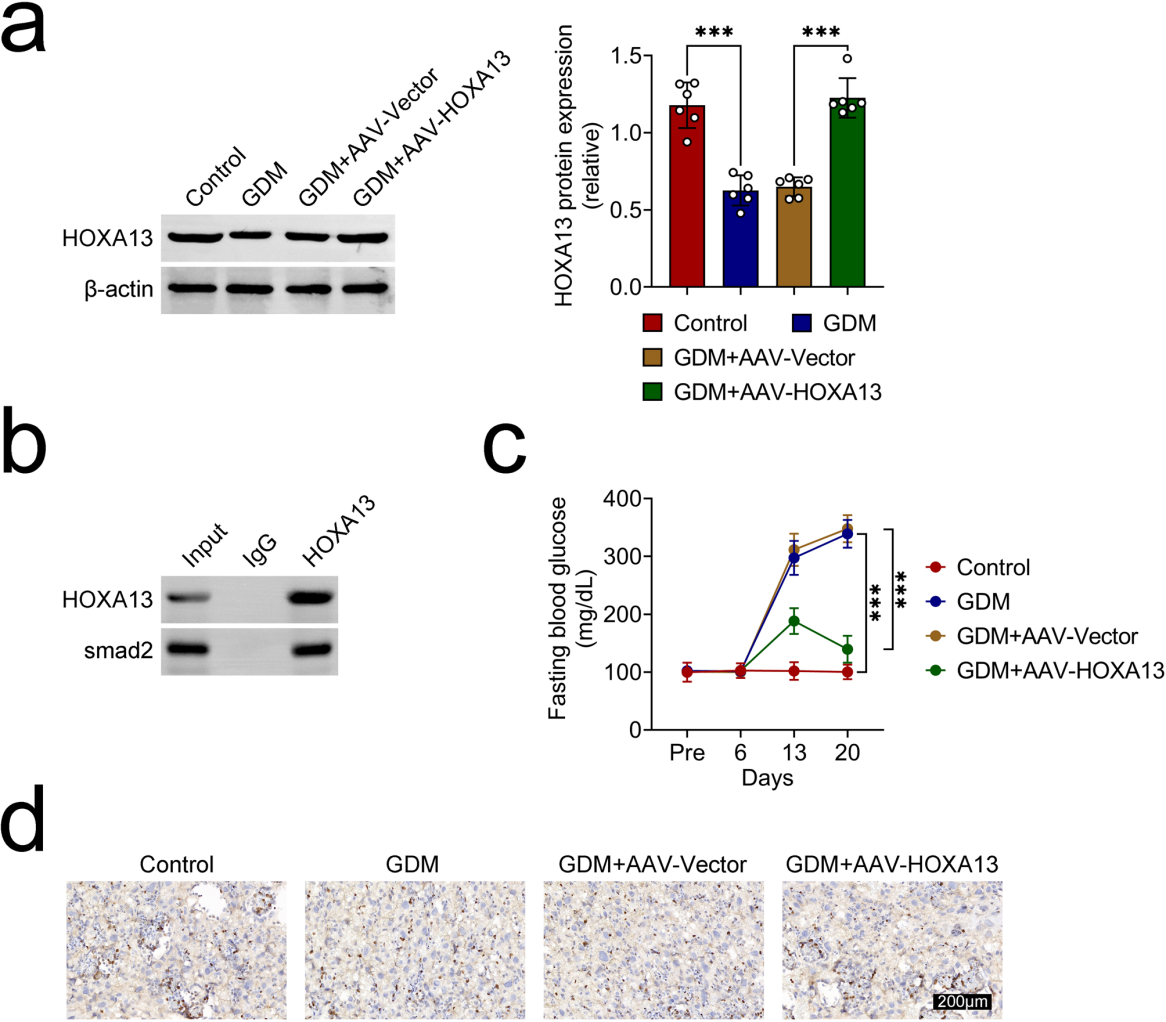
HOXA13 can ameliorate GDM symptoms in vivo. Groups were separated into the Control, GDM, GDM + AAV-Vector and GDM + AAV-HOXA13 group. (**a**) Western blot analysis was conducted to examine the protein levels of HOXA13 in placental tissues. (**b**) The interaction between HOXA13 and smad2 was confirmed through CO-IP assay. (**c**) The fasting blood glucose of rats was detected through the blood glucose meter. (**d**) The Ki67 expression was affirmed through IHC assay. ***p < 0.001

Through in vivo assays, the expression of HOXA13 protein was found to be reduced in the placental tissues of the GDM rat model, but this decrease was reversed following the overexpression of HOXA13 (Fig. 4a). Through CO-IP assay, it was confirmed that HOXA13 can interact with smad2 (Fig. 4b). Besides, the fasting blood glucose was strengthened in GDM rats, but this impact was offset after HOXA13 amplification (Fig. 4c). The Ki67 expression was lessened in GDM rats, but this effect was neutralized after HOXA13 up-regulation (Fig. 4d). In short, HOXA13 can ameliorate GDM symptoms in vivo.

## Discussion

Numerous proteins have been shown to regulate the advancement of GDM. For instance, TDAG51 modulates the SREBP-1/ANGPTL8 pathway in GDM to relieve the damaged lipid metabolism and insulin resistance [18]. In addition, FOXC1 enhances the expression of FGF19, leading to the activation of the AMPK pathway, thus mitigating trophoblast cell damage induced by high glucose in the progression of GDM [19]. MiR-195-5p targets VEGFA to promote endothelial dysfunction in GDM [20]. Moreover, suppression of TXNIP stimulates autophagy to affect cell proliferation and apoptosis in HG-triggered trophoblasts, thereby ameliorating GDM development [21]. Additionally, our prior research indicated that HOXC8 (part of the HOX gene family) has the ability to alleviate the harm caused by high glucose levels on trophoblast cells in GDM [5]. HOXA13 also belongs to the HOX family, and has been clarified to join into some diseases through exhibiting regulatory functions [6–10]. Importantly, hypermethylation of HOXA13 has been confirmed in the placental tissues of individuals with preeclampsia [11]. However, the regulatory impacts of HOXA13 on GDM progression keep indistinct. In this study, HOXA13 was verified to own the declined expression in placental tissues from GDM patients.

Cell proliferation and migration play crucial roles in the progression of GDM [22, 23]. Numerous studies have focused on investigating efficient molecular targets to regulate cell proliferation and movement, ultimately alleviating the progression of GDM. For example, TXNIP contributes to the proliferation and migration abilities of GDM placenta trophoblasts [24]. MiR-137 declines FNDC5 expression to weaken the viability and migration of HTR-8/SVneo cells in GDM [25]. Moreover, MEG3 modulates the AKT pathway to hinder fetal endothelial function in GDM [26]. Additionally, knockdown of BRD4 retards the AKT/mTOR pathway to alleviate the damages of HG-mediated trophoblast cells [27]. Consistent with the findings in previous reports, our research also demonstrated that the cell proliferation and migration abilities of trophoblast cells were attenuated after HG treatment, but these impacts were counteracted after HOXA13 overexpression.

In HG-triggered HTR-8/SVneo cells, smad2 has been proved to be down-regulated, and suppression of smad2 restrains cell proliferation as well as intensifies cell apoptosis [28]. It is of significance to note that the reduction of HOXA13 leads to a decrease in the expression levels of smad2 and smad3 in glioma [29]. However, the relationship between HOXA13 and smad2 in the progression of GDM holds dimness. In a parallel manner, this research also underscored the role of HOXA13 in upregulating smad2 levels, consequently influencing the proliferative and migratory capacities of trophoblast cells stimulated by high glucose. At last, it was further testified that HOXA13 can ameliorate GDM symptoms in vivo (GDM rat model). This work revealed that HOXA13 may influence the proliferation and migration of trophoblast cells by interacting with smad2. This finding shares some similarities with the known mechanisms of action of smad2 regulatory factors (such as TGF-β1, Smad7, etc.) [30, 31]. The role of HOXA13 may lie between these known regulatory factors, influencing the behavior of trophoblast cells by regulating the activity of Smad2.

In conclusion, the study initially revealed that HOXA13 enhances trophoblast cell proliferation and movement induced by high glucose in GDM through its impact on the smad2 pathway. However, certain constraints in this research, such as the absence of clinical trials and the need for additional cellular and animal studies, underscore the necessity for further exploration into the role of HOXA13 in the progression of GDM. This indicates that future investigations will delve deeper into the involvement of HOXA13 in GDM advancement.

**Table 1:** Clinical information

Subjects	NP (*n* = 16)	GDM (*n* = 20)
Age (Y)	28.5 ± 1.51	31.05 ± 2.04
Gestational week (W)	39 ± 0.89	39.2 ± 0.77
Pre-maternal BMI (kg/m2)	19.2 ± 2.96	22.72 ± 2.54***
Maternal weight gain (kg)	12.78 ± 0.67	13.03 ± 0.64
OGTT FPG (mmol/L)	4.83 ± 0.05	5.03 ± 0.08***
OGTT 1hPG (mmol/L)	8.06 ± 0.38	10 ± 0.6***
OGTT 2hPG (mmol/L)	7.68 ± 0.14	7.96 ± 0.32**
HDL (mmol/L)	1.72 ± 0.58	1.83 ± 0.43
LDL (mmol/L)	2.82 ± 0.56	3.09 ± 0.42
Fetal birth weight (g)	3374.05 ± 96.49	3620.01 ± 109.9***
Placental weight (g)	494.09 ± 71.02	486.96 ± 85.67

*p* < 0.01** ; *p* < 0.001***

## Supplementary Information

Below is the link to the electronic supplementary material.

Supplementary Material 1

## Data Availability

All data generated or analyzed during this study are included in this published article. The datasets used and/or analyzed during the present study are available from the corresponding author on reasonable request.
