# Supplementary figures and images for: HOXA13 promotes high glucose-induced trophoblast cell growth and migration during gestational diabetes by regulating the smad2 pathway
Source: Hereditas. 2025 Aug 28;162:176. doi: 10.1186/s41065-025-00542-0 (PMC12395676; doi:10.1186/s41065-025-00542-0)

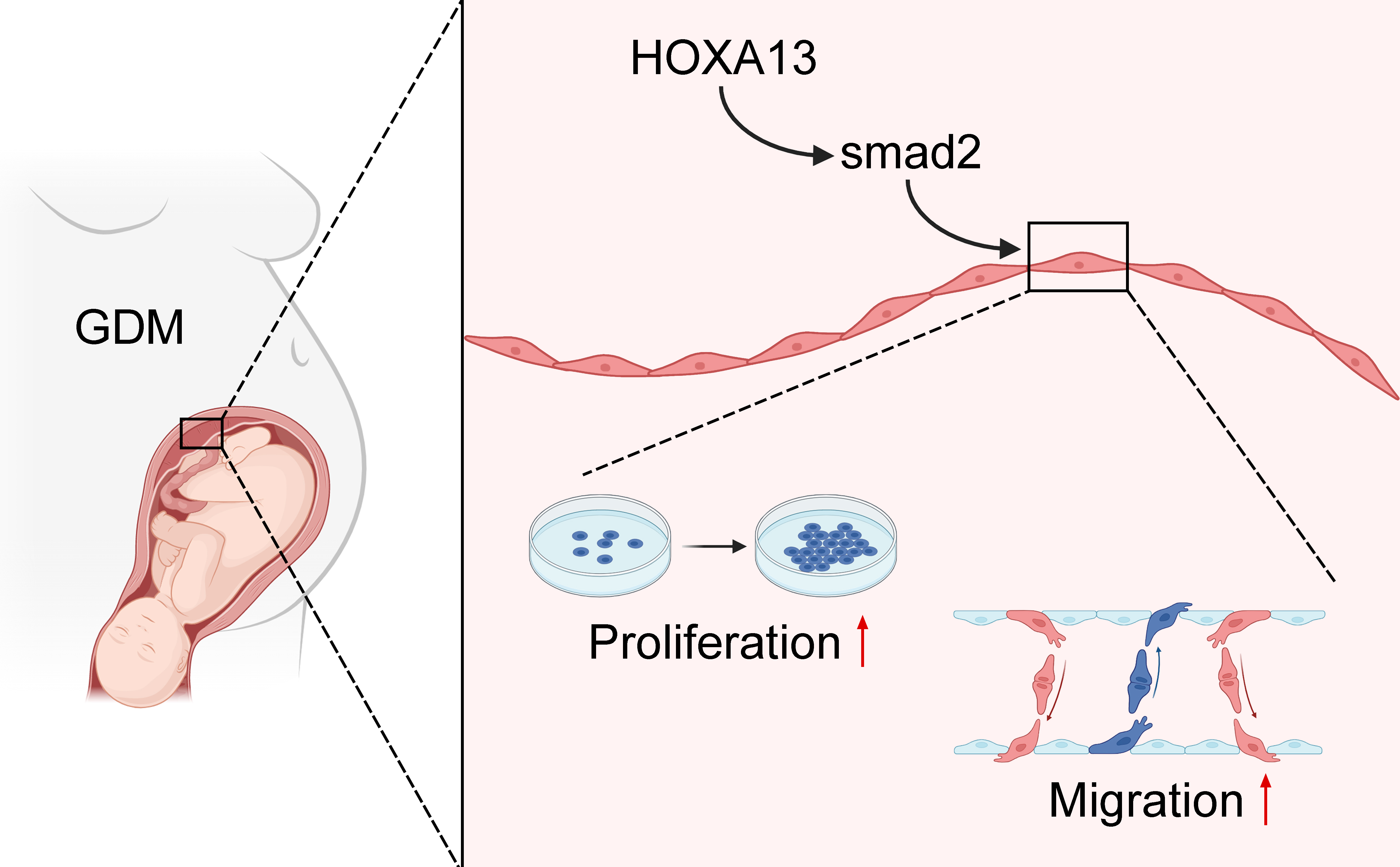

Supplement: Supplementary file 1 — Supplementary Material 1 [file 41065_2025_542_MOESM1_ESM.jpg]
